# Ten Simple Rules for a successful remote postdoc

**DOI:** 10.1371/journal.pcbi.1007809

**Published:** 2020-05-07

**Authors:** Kevin R. Burgio, Caitlin McDonough MacKenzie, Stephanie B. Borrelle, S. K. Morgan Ernest, Jacquelyn L. Gill, Kurt E. Ingeman, Amy Teffer, Ethan P. White

**Affiliations:** 1 Education Department, Cary Institute for Ecosystem Studies, Millbrook, New York, United States of America; 2 Department of Ecology and Evolutionary Biology, University of Connecticut, Storrs, Connecticut, United States of America; 3 Climate Change Institute, University of Maine, Orono, Maine, United States of America; 4 David H. Smith Conservation Research Program, Society for Conservation Biology, Washington, DC, United States of America; 5 Department of Ecology and Evolutionary Biology, University of Toronto, St. George, Ontario, Canada; 6 Department of Wildlife Ecology and Conservation, University of Florida, Gainesville, Florida, United States of America; 7 Informatics Institute, University of Florida, Gainesville, Florida, United States of America; 8 Biodiversity Institute, University of Florida, Gainesville, Florida, United States of America; 9 School of Biology and Ecology, University of Maine, Orono, Maine, United States of America; 10 Department of Ecology, Evolution, and Marine Biology, University of California Santa Barbara, Santa Barbara, California, United States of America; 11 Department of Forest and Conservation Sciences, University of British Columbia, Vancouver, British Columbia, Canada; Carnegie Mellon University, UNITED STATES

## Abstract

Postdocs are a critical transition for early-career researchers. This transient period, between finishing a PhD and finding a permanent position, is when early-career researchers develop independent research programs and establish collaborative relationships that can make a successful career. Traditionally, postdocs physically relocate—sometimes multiple times—for these short-term appointments, which creates challenges that can disproportionately affect members of traditionally underrepresented groups in science, technology, engineering, and mathematics (STEM). However, many research activities involving analytical and quantitative work do not require a physical presence in a lab and can be accomplished remotely. Other fields have embraced remote work, yet many academics have been hesitant to hire remote postdocs. In this article, we present advice to both principal investigators (PIs) and postdocs for successfully navigating a remote position. Using the combined experience of the authors (as either remote postdocs or employers of remote postdocs), we provide a road map to overcome the real (and perceived) obstacles associated with remote work. With planning, communication, and creativity, remote postdocs can be a fully functioning and productive member of a research lab. Further, our rules can be useful for research labs generally and can help foster a more flexible and inclusive environment.

## Introduction

Postdoctoral positions are temporary full-time research positions typically taken between completion of a PhD and the start of a permanent job. These positions almost universally expect postdocs to move and work from the location where they are employed. When research requires specific place-based resources (i.e., field or lab work), it is logical to require postdocs to be working in the same location as lab resources and personnel. However, the proliferation of computational research and virtual communication tools has changed how scientists can conduct science, opening opportunities for postdocs to work remotely, away from the institutions in which they are employed [1]. Relocating for a short-term position (typically 1–3 years) is a substantial burden for early-career researchers. Short-term moves cost time and money, often the equivalent of several months' salary for a postdoc, and can separate people from their support networks. Researchers in long-term relationships or with families may need to live separately or sacrifice career opportunities for partners and support opportunities for children [2–3]. These burdens are magnified for researchers from underrepresented groups [4], e.g., first-generation students who are less likely to have access to financial resources for moving and counteracting the loss of their support networks. Thus, the burden of relocating for short-term postdocs further compounds existing biases that members of these groups face when applying for postdoc positions [5] and contributes to the loss of underrepresented scientists from academia [4].

Working remotely can alleviate these burdens because research activities primarily involving quantitative analysis, modeling, writing, and even some data collection can take place anywhere. In combination with advances in technology (such as video conferencing) that reduce boundaries between remote and in-person interactions, remote postdocs are increasingly possible. However, traditional mindsets of both postdocs and PIs, as well as perceived or existing logistical constraints, can present barriers to making remote postdocs more mainstream.

**Fig 1:**
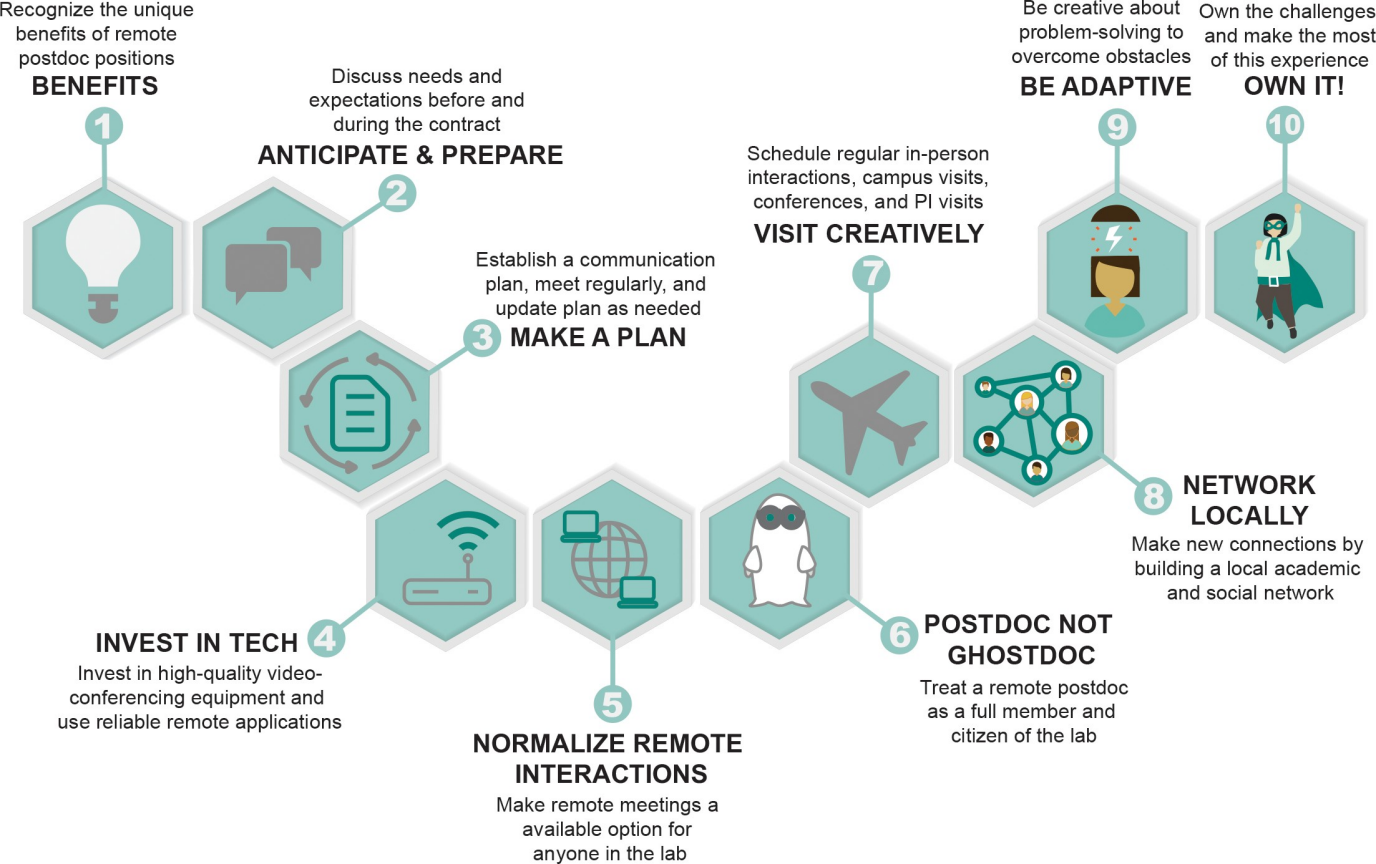
Summary of the Ten Simple Rules in approximately chronological order from advertising for a remote position to establishing a productive working environment and maximizing remote opportunities. PI, principal investigator.

While there are lots of resources for how to work from home effectively (e.g., [6] and [7]), we offer Ten Simple Rules for overcoming challenges and leveraging the unique opportunities presented by remote postdoc positions (Fig 1). We derived these guidelines from our collective experience as remote postdocs and PIs who have mentored them. These rules help illustrate the increasing potential for effective remote postdocs, and adopting them will help improve outcomes for remote postdoc positions, thereby contributing to a healthier culture in science [8]. In addition, adopting many of these practices will also benefit local lab members, improve lab productivity, and facilitate better collaborations among scientists more generally.

## Rule 1: Recognize the benefits of remote postdocs

For many PIs, the benefits of having remote lab members are less familiar than the challenges. However, being open to remote postdocs broadens the applicant pool, fosters inclusive practices, expands the lab network, and allows on-campus mentees to gain experience with remote collaboration. Opening postdoc positions to include remote work can attract more applicants with diverse skills and interests, increasing the chances of finding the best person for the position. Remote postdocs promote inclusion by providing postdoc opportunities to scientists who otherwise would not have access to the mentorship and resources of the lab. These remote postdocs can serve as a conduit of ideas between geographically isolated institutions; remote postdocs foster new collaborations and add value to existing projects with analytical approaches or tools that are not available at the home institution. Furthermore, incorporating remote members into the lab community provides opportunities for the whole lab to engage in and improve remote collaboration skills, which are essential for collaborations with researchers from other institutions and nonacademic partners. Finally, since working remotely can reduce personal burdens and increase support networks, working remotely may help postdocs be more productive than if they moved due to lower stress [9].

## Rule 2: Plan for remote work from the beginning

Prospective remote postdocs and the PIs who mentor them can create the conditions for a successful remote relationship through open and transparent communication throughout the hiring process. Postdocs looking for remote positions should be proactive in approaching potential PIs and negotiating the terms of their remote work. Even if a PI has not explicitly mentioned remote work in their advertisement, they may be open to the possibility of either the current project or a potential proposal. Postdocs can help convince PIs by communicating the benefits of remote postdocs (see Rule 1) and providing evidence of their ability to work independently.

During interviews, discuss the terms of remote work. Be sure to communicate expectations (e.g., communication norms and requirements for fieldwork or mentoring) and limitations (e.g., availability of travel funds) early in the process. What are the institutional policies regarding remote positions? Does the remote position require cross-border consideration (i.e., visas and immigration, which need to be discussed with the hosting institution early)? How often will the postdoc visit? Who pays the costs of visits to campus? Are there temporary housing options available for the postdoc? Asking and answering these questions early will ensure that any legal or institutional issues are addressed and that plans are made to ensure that the postdoc will be effective. If a PI and a potential postdoc are writing a proposal together (e.g., grant or fellowship), openly discuss the costs and benefits of remote postdoc work and budget in travel and housing costs if possible.

Just as with an on-site postdoc, it’s important to establish expectations for mentoring styles and communication (See Rule 3). Early investments in communication and mentorship improve relationships for all members of a lab group (local and remote) by centering transparency and structured collaborations. For remote postdocs, discussing the challenges of working remotely and any associated concerns (of both the PI and postdoc) allows for the development of expectations and a plan for addressing perceived obstacles to communicating, participating in lab activities, mentoring graduate students, and learning new skills remotely.

## Rule 3: Establish a communication plan

A clear communication plan promotes successful mentoring relationships for both locals and remote lab members. It is especially important to establish a communication plan with remote postdocs early in the onboarding process that facilitates the full range of interactions that typically occur within a lab, from casual discussions between lab members to one-on-one meetings, lab meetings, and group discussions. Because each type of communication has different requirements, a suite of tools can be helpful. Email is already a pervasive communication tool that is good for working out complex ideas, giving participants time to reflect and compose their thoughts, and providing a written archive of decisions. However, it doesn’t create a sense of a shared lab space, and many people are already inundated with email. Team-focused collaboration software (we all use Slack) is useful for managing intralab communications in a way that creates a sense of community and facilitates remote equivalents to the kinds of interactions that happen between local lab members. These platforms allow organizing discussions by topic, which allows easy partitioning of questions and discussion by topic and makes it easy for team members to read and engage in discussions on their schedules, which is useful when working in different time zones. For project management and long-term planning, we recommend using shared project management space or shared documents (e.g., project boards on GitHub or checklists on Google Drive) so PIs and postdocs can make sure they agree about priorities, tasks, and progress. It is also important to revisit your communication plan at set intervals (e.g., semiannual reviews) to make sure established mechanisms are working for both the remote and local lab members, because plans, preferences, and schedules may change throughout a postdoctoral position.

## Rule 4: Invest in and use video conferencing

As remote postdocs and PIs work to create a solid, functional, and dynamic communication plan (Rule 3), investing in the hardware, software, and practice of video conferencing will both support that communication and open the whole lab to dynamic, inclusive meetings (Rule 5). Leveraging modern technology to conduct regular real-time meetings will allow remote postdocs to build relationships with their lab, facilitate brainstorming across the whole lab community, and obtain the advising and support they need to be professionally successful. Text-based communication (e.g., emails and collaboration platforms such as Slack) has many advantages, but it can be problematic in many contexts because it is easy to misconstrue tone due to the lack of visual and auditory cues. It can also be cumbersome when dealing with complex issues or multiperson discussions. Adding video or audio allows for more subtle communication, including body language and tone, and improves the establishment of rapport between the remote postdoc and their PI and lab mates.

**Fig 2:**
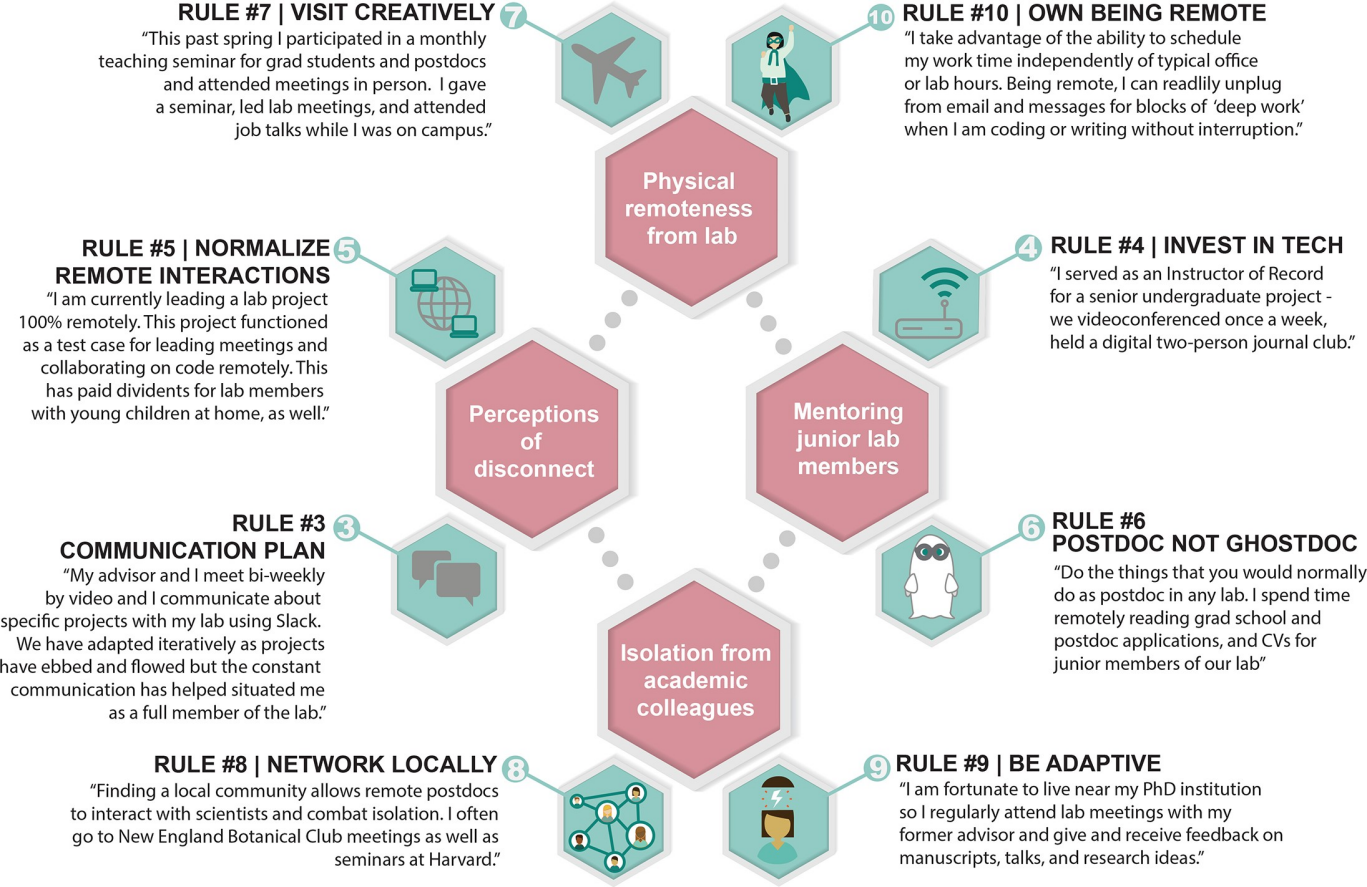
Simple rules for remote postdocs (green) applied to several common postdoc situations (pink). Quotes are from authors with direct experience of the associated rule and situation.

Video conferencing can be used to replace some in-person (one-on-one) meetings, and there should be support for video attendance for all lab and project meetings (see Fig 2 for a real-world example). Lab processes should be developed to provide full value to video participants (such as remote postdocs). For example, when local participants present slides during meetings, they should share their slides by screen sharing through video conferencing software rather than pointing a camera at the screen. Many video conference platforms (e.g., Skype for Business and Zoom) allow presenters to share their screen with the other people on the call. Remotely shared screens enable lab members to conduct practice talks, troubleshoot code in real-time, discuss data, or live-collaborate on papers. Some platforms (e.g., Zoom) also support”conference rooms” that anyone can join at any time and “breakout rooms” that allow group video conferences to split into concurrent sessions to facilitate subgroup discussions in virtual spaces.

While good video conferencing can be a great way to interact, bad video conferencing can be frustrating due to poor sound, poor video, and other technological issues. Therefore, it is important to have access to good video conferencing setups for both the postdoc and the lab. Invest in a video conferencing system for the lab (good built-in systems are surprisingly affordable) or find and schedule rooms at your university that are equipped for remote participation. Become familiar with university resources for teleconferencing, including license agreements for video conference software packages (you might have free access as an employee and not know it). Wired internet access is often more stable than a wireless connection, especially in remote locations. Finally, for remote meetings (as with in-person meetings), it is critical to establish and follow an agenda to keep conversations from getting sidetracked.

## Rule 5: Normalize remote interactions and cultivate digital spaces for the entire lab

Digital collaboration on writing, coding, and discussion is increasingly central to productive research. While digital collaboration tools are essential to remote collaboration, they are also useful for local collaboration. Even when collaborators work within the same institution, they may have different schedules, obligations, or frequent travel requirements that can prevent them from coming into the lab. Digital spaces allow lab members to interact seamlessly regardless of remote or local status. For example, this manuscript was written entirely through a remote collaboration using email, Slack, and Google Drive.

It is vital to normalize remote interactions and collaboration tools throughout the lab, not just for remote postdocs. Integrating digital spaces into the everyday lab practices reduces the differences between local and remote members and lowers the barriers for interaction. Electronic lab or research notebooks, wikis (we have used GitHub wikis and Open Science Framework), project management software (we have used GitHub and Trello), online office software (we have used Google Docs, Sheets, and Slides), and cloud storage (we have used Google Drive, Zenodo, and figshare) improve institutional memory, create an archive of activities, help with project management, and ensure long-term, secure data storage, therefore providing many advantages in addition to supporting remote work. Remote participation can be useful for local individuals as well, allowing for work to continue when traveling or working from home (see Fig 2 for a real-world example), some examples include: the use of institutional virtual private networks (VPNs), remote access to computing clusters, and interlibrary-loan scanning services. Fostering an environment of inclusion and support for remote work makes remote postdocs less of an outlier and also improves quality of life for local lab members as well.

## Rule 6: Remote postdocs are full members of the lab

While there may be constraints or differences in your mentoring approach with a remote postdoc, PIs should not think of them as separate from the rest of the lab. Remote postdocs should be included when you share opportunities, celebrate accomplishments, and plan lab activities, just as with local lab participants. This will model good collaborative behavior for the lab and normalize remote interactions that will be increasingly prevalent as students and trainees progress in their careers.

One of the central roles played by postdocs is their vital role as mentors of more junior lab members [10]. Working remotely doesn’t mean postdocs can’t participate in lab discussions, build lab community, or provide direct mentorship to junior lab members. Giving feedback on manuscripts, chapter drafts, and code works just as effectively remotely (see Fig 2 for a real-world example). By using video conferencing (Rule 4) and text-based communication tools (Rules 3 and 5), remote postdocs can lead group projects, mentor graduate and undergraduate students, and connect with lab members. Another way to be a full member of the lab is to actively take part in day-to-day lab duties that are possible to do remotely, which can include planning lab celebrations, organizing lab meetings, and sharing notes in journal clubs.

## Rule 7: A little in-person interaction goes a long way; maximize it by being creative

Create opportunities for remote postdocs to visit the home campus; leverage travel around conferences and workshops to support lab reunions and face-to-face mentoring. Off-site in-person meetings at conferences can provide remote postdocs and PIs time for intensive work together away from the distractions of the home campus. Similarly, when remote postdocs attend conferences or workshops with other lab mates, community and collaboration are built in the lab through face-to-face interactions.

One to two in-person, on-campus interactions a year can provide a lot of value for remote postdocs and their home labs. Both PIs and postdocs can maximize the benefits of this time on campus. Create opportunities for professional growth, but don’t underestimate the social aspect of visits to the home institution (see Fig 2 for a real-world example). Informal gatherings are as important as giving seminars and setting up professional meetings and can help postdocs rest and recharge to take full advantage of a short visit. Minimize opportunity costs by inviting your remote postdoc to give a department seminar, nominating them to speak at an on-campus symposium, or asking them to serve on a committee that meets only a few times during the semester or can handle remote participation (e.g., honors thesis committees). With some foresight, you can leverage these opportunities to subsidize a campus visit and then strategically schedule lab meetings, social activities, and networking during the visit. Search through the calendars of on-campus groups and adjacent departments to identify seminars, lunches, and meetings that may add value to your postdoc’s visit, even if they are “off the beaten path.” Will there be a Story Collider show or a reading in town by a popular science communicator? Is your campus’s women in STEM group hosting a workshop for allies? Does the college town bakery have fresh doughnuts at the local farmer's market on certain mornings?

Facilitating a visit for a remote postdoc and helping them fill their schedule with a mix of academic, social, and networking opportunities can bring the entire lab closer together and sharpen your skills for inviting and hosting campus visitors in the future, from senior researchers to faculty candidates.

## Rule 8: Actively work to combat isolation

As members of an often-neglected career stage (i.e., few institutions have postdoc-centered support services), postdocs often feel isolated. Distance from a home institution can exacerbate feelings of loneliness, imposter syndrome, and the stress and uncertainty of the job market. For many, the postdoc stage is contemporaneous with new challenges like starting a family, caring for aging parents, financial instability, and the loss of strong social connections built during college or graduate school. Therefore, it is vital to combat isolation, prioritize social support, and practice self-care.

One way for remote postdocs to reduce isolation is to cultivate and maintain connections in their local community. Remote postdocs can develop or maintain an academic community in the location where they are living. Interacting with a relevant lab at a local institution, even informally, provides a platform to talk about your work with a knowledgeable audience, to receive feedback on writing or presentations, to discuss papers, and to practice mentoring graduate students. Depending on the local institution, it may be possible for the remote postdoc to obtain access to workspace, network, library, and recreational facilities (see Fig 2 for a real-world example). Local institutions may also have seminar series that are relevant to your discipline, offering inspiration and the chance to cultivate relationships with potential collaborators.

Remote interactions offer another avenue to combat isolation, (for example, by including remote postdocs in lab projects that involve regular communication with other lab members). Remote postdocs can lead lab meetings, participate in journal clubs, offer “office hours,” review the writing and coding of other lab members, serve on committees, and even participate in social events (either calling into the event itself or helping in the planning process). Fun slack channels or group texts can stand in for incidental water-cooler interactions or opportunistic group lunches. Combatting isolation should be a priority for both postdocs and PIs. While the quality of life benefits are worthwhile in their own right, reduced stress can also lead to better work [9].

## Rule 9: Develop adaptive problem-solving skills

There are clear benefits that come with remote postdocs, but there are also real tradeoffs. Applying the rules as we describe here can manage some of these tradeoffs. However, each situation is different, and novel challenges or obstacles are likely to arise for both the PI and the postdoc. Being adaptive to novel obstacles starts with an open communication channel (see Rules 2 and 3). Discuss challenges as they emerge and before they become intractable. Both the PI and the postdoc should be prepared to be flexible in their thinking and adaptive in how they deal with challenges and obstacles or even exciting opportunities that might arise. For the postdoc, actively engaging their mentor as they navigate challenging or unexpected situations resulting from working remotely can be important. This is broadly applicable advice, as local postdocs may also feel pressure to take care of challenges on their own, but turning to a mentor for guidance is not a sign of weakness and can help everyone more quickly identify and fix issues that arise from remote work.

Second, be creative about overcoming distance challenges. For example, Caitlin McDonough MacKenzie successfully mentored an undergraduate project and led a journal reading group solely via remote interactions (Fig 2). Creative problem solving means that you don’t have to replicate the standard postdoc position to gain many of the same experiences. Finally, take advantage of existing academic relations or networks that can provide creative opportunities to fill the distance between you and your remote lab community (see Fig 2 for a real-world example). With open communication and creativity, the postdoc and the PI can develop dynamic problem-solving skills that will benefit both throughout their careers.

## Rule 10: Own and promote mentoring or being a remote postdoc

Embracing remote work as a legitimate working model will require a shift in how we think about postdoc research. In our experience, the perception that remote postdocs contribute less and are inaccessible to their labs is both common and incorrect. We should actively confront this and other negative perceptions to offer a new mindset for remote postdocs, their mentors, and the broader academic community. Doing so involves openly discussing remote postdoctoral work, and ways to accomplish it effectively (including the rules outlined in this paper) and encouraging colleagues advertising for postdocs to actively consider remote applicants. With good planning, integration, confidence, and communication, fantastic science and scientists can emerge from remote postdoc arrangements!

## Conclusions

We have provided a set of guidelines for facilitating successful remote postdoctoral experiences (Fig 1). The core of this advice is to treat this person as you would a coauthor/collaborator/co-PI from another institution (e.g., [11]) and embed tools that facilitate remote collaboration as a core component of how a lab operates. Because so much modern collaboration happens virtually or involves relatively few in-person meetings (e.g., working groups), the tool kits needed to support communication and remote postdoc positions already exist. While implementing our guidelines will require some effort, the benefits of doing so will extend far beyond the remote postdoctoral researcher. Following these rules and tailoring them to each lab’s specific circumstances will support the group's ability to interact with colleagues at other institutions, improve communication among lab members (including local ones), support introverted lab members, and provide flexibility for lab members juggling multiple obligations. Indeed, many of these approaches improve collaboration among lab members in general, allow parents to work around childcare responsibilities, and support the participation of lab members with illnesses or disabilities that make commuting to campus difficult [8]. The time has come to view remote postdoc research as part of a diversity of viable models for employing and training postdoctoral researchers.

## References

[1] Ríos-Saldaña CA, Delibes-Mateos M, Ferreira CC. Are fieldwork studies being relegated to second place in conservation science? Global Ecology and Conservation. 2018 4 1;14:e00389.

[2] Jacobs JA. Presidential address: The faculty time divide. In Sociological Forum. 2004 3 1 (Vol. 19, No. 1, pp. 3–27). Kluwer Academic Publishers-Plenum Publishers.

[3] Rivera LA. When two bodies are (not) a problem: Gender and relationship status discrimination in academic hiring. American Sociological Review. 2017 12;82(6):1111–38.

[4] Grogan KE. How the entire scientific community can confront gender bias in the workplace. Nature Ecology and Evolution. 2019 1;3(1):3 10.1038/s41559-018-0747-4 30478306

[5] Eaton AA, Saunders JF, Jacobson RK, West K. How Gender and Race Stereotypes Impact the Advancement of Scholars in STEM: Professors’ Biased Evaluations of Physics and Biology Post-Doctoral Candidates. Sex Roles. 2019:1–5.

[6] Frater PN, Sullivan LL. Six tips for happy, productive remote working. Science. 2018 10.1126/science.caredit.aaw2750

[7] Payne D. The pros and cons of mentoring by Skype. Nature, 2018 10.1038/d41586-018-05794-7

[8] Maestre FT. Ten simple rules towards healthier research labs. PLoS Comput Biol. 2019 4 11;15(4):e1006914 10.1371/journal.pcbi.1006914 30973866 PMC6459491

[9] Waltman J, Sullivan B. Creating and supporting a flexible work-life environment for faculty and staff. Effective Practices for Academic Leaders. 2007 2(2):1.

[10] Feldon DF, Litson K, Jeong S, Blaney JM, Kang J, Miller C, et al Postdocs’ lab engagement predicts trajectories of PhD students’ skill development. Proceedings of the National Academy of Sciences. 2019 116(42): 20910–20916. 10.1073/pnas.1912488116 PMC6800364 31570599

[11] Frassl MA, Hamilton DP, Denfeld BA, de Eyto E, Hampton SE, Keller PS. Ten simple rules for collaboratively writing a multi-authored paper. PLoS Comput Biol 2018 14(11):e1006508 10.1371/journal.pcbi.1006508 30439938 PMC6237291

